# Supplementary material for: Age of Red Cells for Transfusion and Outcomes in Patients with ARDS
Source: J Clin Med. 2022 Jan 4;11(1):245. doi: 10.3390/jcm11010245 (PMC8745782; doi:10.3390/jcm11010245)
Supplement: Supplementary file 1 [file jcm-11-00245-s001.zip › jcm-1486465-supplementary.pdf]

Additional File 1

Age of Red Cells for Transfusion and Outcomes in Patients with ARDS

Authors

J. A. Graw, V. Bünger, L. Materne, A. Krannich, F. Balzer, R. C. Francis, A. Pruß, C. Spies, W. M. Kübler, S. Weber-Carstens, M. Menk, O. Hunsicker

Table of Contents

Supplemental Methods 2
Definition of "failure-free days" composites 2
Ventilator-free days composite (VFDs) 2
Sedation-free days composite 2
Renal replacement therapy-free days composite 3
Vasopressor-free days composite 3
Data sources 4
Supplemental Tables 5
Table S1: Baseline characteristics of the patients that had received at least one unit of PRBCs with a storage age >28 days and patients who only received PRBCs with a storage age ≤28 days 5
Table S2. Transfusion characteristics of the patients that had received at least one unit of PRBCs with a storage age >28 days and patients who only received PRBCs with a storage age ≤28 days. 5
Supplemental Figures 7
Figure S1: Censoring between the short-term and long-term storage group. 7
Figure S2: Transfusion characteristics of patients that had received at least one unit of PRBCs with a storage age >28 days and patients who only received PRBCs with a storage age ≤28 days 8
Figure S3: Cumulative incidence curve of RRT-free days composite between patients that had received at least one unit of PRBCs with a storage age >28 days and patients who only received PRBCs with a storage age ≤28 days. 9
References 10

Supplemental Methods

Definition of "failure-free days" composites

"Failure-free days" composites were assessed defined and analyzed according to the most recent recommendations [1]. Briefly, except for the ECMO-free days composite, we defined the ARDS onset as start time, used a timeframe of 28 days, assigned all 28-day non-survivors 0 failure-free days irrespective if the event of interest occurred before, and censored observations after 28 days. In the following a detailed definition of each "failure-free days" composite is presented.

Ventilator-free days composite (VFDs)

The ventilator-free days composite was defined according to the most recent recommendations by Yehya et al [1].

Start time	Day of ARDS onset.
Timeframe	28 days.
Successful weaning from mechanical ventilation	Extubation >48 hours without reintubation or >48 hours off of positive pressure in patients with tracheostomy.
Interval extubation	We counted from the day of final successful extubation if there were repeat intubation episodes in the first 28 days.
Non-invasive support and tracheostomies	Non-invasive support was not counted and tracheostomies were treated as other invasive ventilation.
Value for extubated decedent	All 28-day non-survivors were counted 0 VFDs, irrespective of their intubation status, and censored observations after 28 days.

Sedation-free days composite

Start time	Day of ARDS onset.
Timeframe	28 days.
Successful weaning from sedation	Richmond Agitation-Sedation Scale (RASS) 0 or -1. [Successful weaning from sedation was defined as published previously [2, 3].]
Interval weaning from sedation	We counted from the day of final RASS 0 or -1 if there

	were repeat episodes of need for a deeper sedation in the first 28 days.
Value for decedent after successful weaning from sedation	All 28-day non-survivors were counted 0 sedation-free days, irrespective of successful weaning from sedation before and censored observations after 28 days.

Renal replacement therapy-free days composite

Start time	Day of ARDS onset.
Timeframe	28 days.
Successful weaning from renal replacement therapy	Stopping renal replacement therapy without re-initiation up to day 28.
Interval weaning from renal replacement therapy	We counted from the day of finally stopping renal replacement therapy if there were repeat episodes of re-initiation of renal replacement therapy in the first 28 days.
Value for decedent after weaning from renal replacement therapy	All 28-day non-survivors were counted 0 renal replacement therapy-free days, irrespective of successful weaning from renal replacement therapy before and censored observations after 28 days.

Vasopressor-free days composite

Start time	Day of ARDS onset.
Timeframe	28 days.
Successful weaning from vasopressors	Stopping vasopressors without re-initiation up to day 28.
Interval weaning from vasopressors	We counted from the day of finally stopping vasopressors if there were repeat episodes of starting vasopressors again in the first 28 days.
Value for decedent after weaning from vasopressors	All 28-day non-survivors were counted 0 vasopressor-free days, irrespective of successful weaning from vasopressors before and censored observations after 28 days.

Data sources

Data on patients' demographics and comorbidities were extracted from the hospital data management system (SAP, Walldorf, Germany). Data on admission scores, ARDS characteristics, ARDS treatment, rescue therapies, medications, ventilation parameters, blood transfusions, RRT, and laboratory parameters such as hemoglobin measurements were extracted from the electronic intensive care unit data management system in use at the hospital (COPRA 5, Sasbachwalden, Germany). PRBC storage data was obtained from the transfusion database maintained by Institute of Transfusion Medicine, Charité - Universitätsmedizin Berlin.

Supplemental Tables

Table S1: Baseline characteristics of the patients that had received at least one unit of PRBCs with a storage age >28 days and patients who only received PRBCs with a storage age ≤28 days.

Characteristic	Oldest PRBC unit ≤28 days (n=99)	Oldest PRBC unit >28 days (n=184)	P-value
**Age** (years)	55.00 [38.50, 65.50]	57.00 [44.75, 68.00]	0.394
**Male sex**, n (%)	58 (58.6)	118 (64.1)	0.430
**Body mass index** (kg/cm)	28.68 [24.96, 33.60]	26.62 [23.66, 31.31]	0.029
**Charlson comorbidity index**	3.00 [0.50, 5.00]	3.00 [1.00, 6.00]	0.555
**Chronic kidney disease**, n (%)	9 (9.1)	26 (14.1)	0.299
**Immunocompromised**, n (%)	23 (23.2)	43 (23.4)	1.000
**SOFA at ARDS onset**	10.00 [8.00, 13.00]	12.00 [9.00, 14.00]	0.019
**SAPS II at ARDS onset**	46.00 [34.50, 64.50]	55.00 [40.00, 67.25]	0.029
**RASS at ARDS onset**	-4.50 [-5.00, -4.00]	-5.00 [-5.00, -4.00]	0.237
**Chronic lung disease**, n (%)	18 (18.2)	45 (24.5)	0.289
**Mechanical ventilation before admission** (days)	1.00 [0.00, 3.00]	1.00 [0.00, 4.00]	0.138
**ARDS severity**, n (%)			0.294
Mild	3 (3.0)	14 (7.6)	
Moderate	23 (23.2)	43 (23.4)	
Severe	73 (73.7)	127 (69.0)	
**ARDS etiology**, n (%)			0.207
Pneumonia	56 (56.6)	97 (52.7)	
Aspiration	25 (25.3)	36 (19.6)	
Sepsis	4 (4.0)	22 (12.0)	
Pancreatitis	2 (2.0)	6 (3.3)	
Other	12 (12.1)	23 (12.5)	
**Ventilation parameters after initial optimization**			
PaO2:FiO2 (mmHg)	154.02 [107.40, 219.48]	161.77 [112.89, 218.99]	0.778
Oxygenation index	15.23 [9.55, 22.41]	14.36 [9.10, 22.87]	0.691
PEEP (cm H2O)	16.05 [13.90, 20.00]	16.40 [13.40, 18.30]	0.498
Driving pressure (cm H2O)	14.89 [12.46, 18.01]	15.45 [12.90, 18.21]	0.755
Tidal volume (ml/kg PBW)	6.69 [5.84, 7.50]	6.39 [5.55, 7.55]	0.469
Respiratory rate (breaths/min)	20.50 [18.00, 24.00]	21.00 [19.00, 24.75]	0.840
Compliance (ml/cm H2O)	36.75 [27.62, 49.32]	34.80 [27.15, 46.50]	0.545
**Rescue therapy**			
Inhaled nitric oxide, n (%)	71 (71.7)	117 (63.6)	0.212
Prone positioning, n (%)	65 (65.7)	119 (64.7)	0.972
**Septic shock**, n (%)	37 (37.8)	83 (45.9)	0.239
**Lactate** (mg/dl)	14.50 [10.00, 25.00]	17.00 [10.75, 31.00]	0.120
**RRT**, n (%)	60 (60.6)	110 (59.8)	0.994

Definition of abbreviations: SOFA = Sequential Organ Failure Assessment, SAPS = Simplified Acute Physiology Score, RASS = Richmond Agitation-Sedation Scale, PEEP = Positive End-Expiratory Pressure, RRT = Renal replacement therapy.
Data are expressed as median [25%, 75% quartiles] or frequencies (%), as appropriate. P-values were calculated using the exact Wilcoxon-Mann-Whitney test and the Fisher's exact test, as appropriate

Table S2. Transfusion characteristics of the patients that had received at least one unit of PRBCs with a storage age >28 days and patients who only received PRBCs with a storage age ≤28 days.

**Characteristic**	**Oldest PRBC unit ≤28 days** (n=99)	**Oldest PRBC unit >28 days** (n=184)	**P-value**
**Hemoglobin on admission** (g/dl)	10.50 [9.30, 12.05	10.15 [9.20, 11.83]	0.325
**Transfusion threshold*** (g/dl)	8.20 [7.53, 9.15]	8.20 [7.60, 8.90]	0.824
**PRBC units transfused per patient with the first 14 days** (number)	3.00 [2.00, 6.00]	6.00 [3.00, 10.00]	<0.001
**PRBC units transfused per patient with the first 28 days** (number)	4.00 [2.00, 8.00]	6.00 [3.00, 12.00]	<0.001
**Oldest PRBC unit per patient** (days)	21.37 (5.52)	35.96 (3.89)	<0.001
**Time to first PRBC transfusion** (hours)	16.25 [12.11, 19.29]	15.85 [11.63, 19.00]	0.501
**Patients receiving transfusion of other blood components,** n (%)			
Platelets	18 (18.2)	57 (31.0)	0.029
Fresh frozen plasma	70 (70.7)	124 (67.4)	0.661

Definition of abbreviations: PRBC = packed red blood cells.

Data are expressed as mean (SD), median [25%, 75% quartiles] or frequencies (%), as appropriate. P-values were calculated using the t-test, the exact Wilcoxon-Mann-Whitney test and the Fisher's exact test, as appropriate. * An individual hemoglobin threshold for RBC transfusion was calculated for each patient identifying the lowest hemoglobin concentration during a period of 6 h prior to transfusion for each RBC unit during the first 14 days after ARDS onset and then averaging the lowest hemoglobin concentrations over the number of transfused RBC units, as reported previously [25].

Supplemental Figures

Figure S1: Censoring between the short-term and long-term storage group. An equal distribution of censoring was checked using Kaplan–Meier estimates showing the cumulative proportion of patients censored with respect to the primary endpoint (A) and RRT-free days composite (B) within 28 days after onset of ARDS between the short-term and long-term storage group.

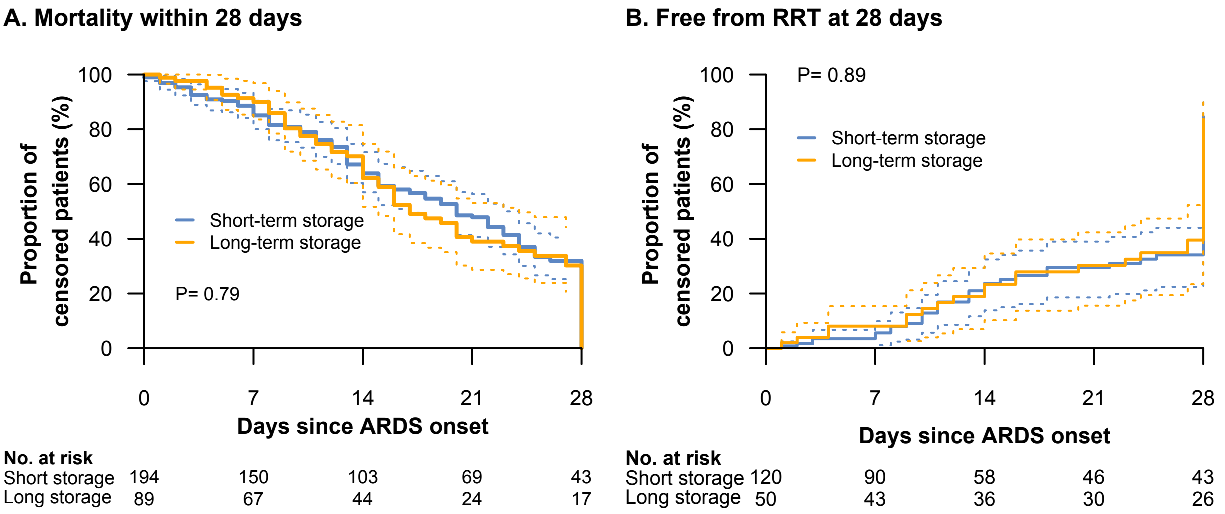

Figure S2: Transfusion characteristics of patients that had received at least one unit of PRBCs with a storage age >28 days and patients who only received PRBCs with a storage age ≤28 days. The hemoglobin concentrations at ARDS onset (A), the transfusion threshold (pre-transfusion hemoglobin) (B), the time to transfusion of the first PRBC unit (C), the number of transfused RBC units within 14 days of ARDS therapy (D), and the distribution of the age of the oldest PRBC unit in both groups (E) are presented. The mean storage (and SD) in each group and the mean difference between the groups is indicated. *** $P<0.001$.

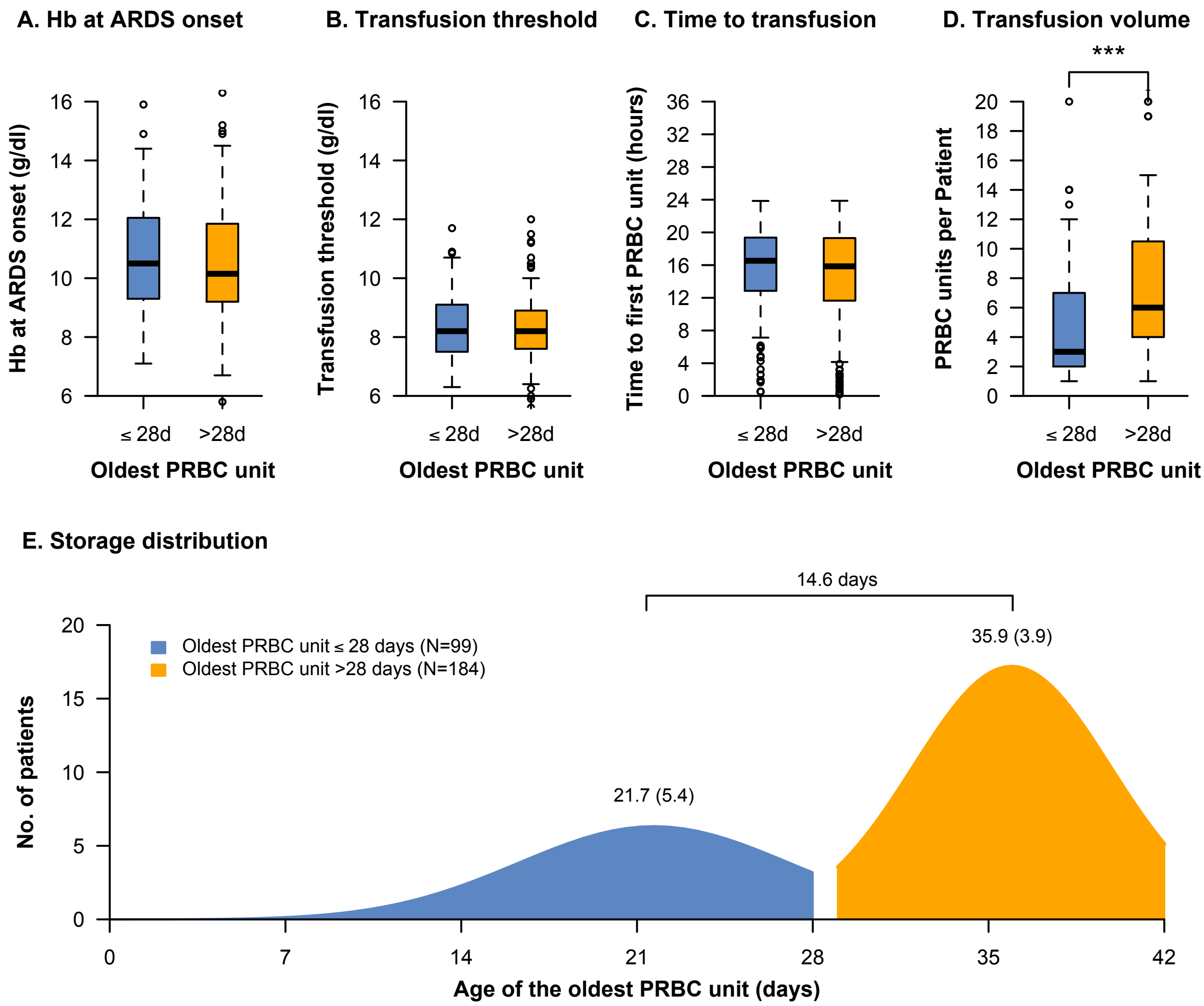

Figure S3: Cumulative incidence curve of RRT-free days composite between patients that had received at least one unit of PRBCs with a storage age >28 days and patients who only received PRBCs with a storage age ≤28 days. For each curve 95% confidence intervals (dotted lines) are shown. The subdistribution hazard ratio (SHR) with 95% confidence intervals is provided. The SHR is calculated from a competing risk regression providing the chance of patients that had received at least one unit of PRBCs with a storage age >28 days compared to patients who only received PRBCs with a storage age ≤28 days for weaning from RRT accounting for the existence of the alternative outcome of death. The SHR was adjusted for the number of PRBC units transfused per patient with the first 14 days after ARDS onset.

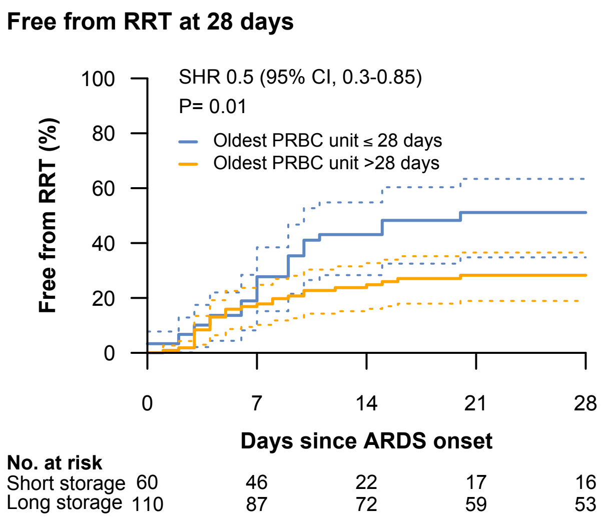

References

1. Yehya N, Harhay MO, Curley MAQ, Schoenfeld DA, Reeder RW, (2019) Re-appraisal of Ventilator-free Days in Critical Care Research. Am J Respir Crit Care Med
2. Devlin JW, Skrobik Y, Gelinas C, Needham DM, Slooter AJC, Pandharipande PP, Watson PL, Weinhouse GL, Nunnally ME, Rochwerg B, Balas MC, van den Boogaard M, Bosma KJ, Brummel NE, Chanques G, Denehy L, Drouot X, Fraser GL, Harris JE, Joffe AM, Kho ME, Kress JP, Lanphere JA, McKinley S, Neufeld KJ, Pisani MA, Payen JF, Pun BT, Puntillo KA, Riker RR, Robinson BRH, Shehabi Y, Szumita PM, Winkelman C, Centofanti JE, Price C, Nikayin S, Misak CJ, Flood PD, Kiedrowski K, Alhazzani W, (2018) Clinical Practice Guidelines for the Prevention and Management of Pain, Agitation/Sedation, Delirium, Immobility, and Sleep Disruption in Adult Patients in the ICU. Crit Care Med 46: e825-e873
3. Muller A, Weiss B, Spies CD, Leitliniengruppe S, (2015) ["Symptomatic Treatment of Delirium, Anxiety and Stress, and Protocol Based Analgesia, Sedation and Management of Sleep in Intensive Care Patients"]. Anasthesiol Intensivmed Notfallmed Schmerzther 50: 698-703
